# Genetics of Obesity Traits: A Bivariate Genome-Wide Association Analysis

**DOI:** 10.3389/fgene.2018.00179

**Published:** 2018-05-16

**Authors:** Yili Wu, Haiping Duan, Xiaocao Tian, Chunsheng Xu, Weijing Wang, Wenjie Jiang, Zengchang Pang, Dongfeng Zhang, Qihua Tan

**Affiliations:** ^1^Department of Epidemiology and Health Statistics, Public Health College, Qingdao University, Qingdao, China; ^2^Qingdao Municipal Center for Disease Control and Prevention, Qingdao, China; ^3^Epidemiology and Biostatistics, Department of Public Health, University of Southern Denmark, Odense, Denmark; ^4^Unit of Human Genetics, Department of Clinical Research, University of Southern Denmark, Odense, Denmark

**Keywords:** obesity, signaling by G protein-coupled receptor, olfactory transduction, bivariate genome-wide association study, twin study

## Abstract

Previous genome-wide association studies on anthropometric measurements have identified more than 100 related loci, but only a small portion of heritability in obesity was explained. Here we present a bivariate twin study to look for the genetic variants associated with body mass index and waist-hip ratio, and to explore the obesity-related pathways in Northern Han Chinese. Cholesky decomposition model for 242 monozygotic and 140 dizygotic twin pairs indicated a moderate genetic correlation (*r* = 0.53, 95%CI: 0.42–0.64) between body mass index and waist-hip ratio. Bivariate genome-wide association analysis in 139 dizygotic twin pairs identified 26 associated SNPs with *p* < 10^−5^. Further gene-based analysis found 291 nominally associated genes (*P* < 0.05), including *F12, HCRTR1, PHOSPHO1, DOCK2, DOCK6, DGKB, GLP1R, TRHR, MMP1, GPR55, CCK*, and *OR2AK2*, as well as 6 enriched gene-sets with FDR < 0.05. Expression quantitative trait loci analysis identified rs2242044 as a significant cis-eQTL in both the normal adipose-subcutaneous (*P* = 1.7 × 10^−9^) and adipose-visceral (*P* = 4.4 × 10^−15^) tissue. These findings may provide an important entry point to unravel genetic pleiotropy in obesity traits.

## Introduction

Obesity is a worldwide epidemic associated with increased morbidity and mortality, and greatly contributes to the global disease burden (Fall and Ingelsson, 2014). The evidence from family and twin studies have showed that around 40–70% (Sandholt et al., 2012) of the obesity variation in human was attributed to genetic factors. Searching for related susceptibility loci would help to better understand the molecular mechanisms and biological pathways of obesity.

Usually obesity is approximately assessed by anthropometric measurements including body mass index (BMI) and waist-hip ratio (WHR), because of its low cost of efficiency (Fall and Ingelsson, 2014). Previous genome-wide association studies (GWAS) on these anthropometric measurements using univariate analysis identified more than 100 related loci, but only a small portion of heritability in obesity was explained by those genetic variants (Fall and Ingelsson, 2014; Locke et al., 2015; Shungin et al., 2015). Since complex disease is contributed from many genetic factors, theoretically univariate GWAS is less powerful for identifying the potential genetic correlation in correlated traits such as BMI and WHR. Therefore, it is hard to identify the pleiotropic genes associated with a set of correlated phenotypes that play a central role in the pathogenesis (Allison et al., 1998; Lu et al., 2016). Bivariant or multivariate association studies, which consider two or more phenotypes simultaneously, could help to address this issue. They not only could find pleiotropic genes, but also could relieve multiple testing problems. These studies have successfully identified susceptibility genes for several complex traits, including Kashin-Beck disease, heart rate and low density lipoprotein subfractions (Melton et al., 2010; Shim et al., 2015; Hao et al, 2016). However, limited bivariate and multivariate GWA analyses of obesity-related phenotypes have been reported.

Human twins are two offspring produced during one pregnancy. Monozygotic (MZ) twin share 100% of their genetic background, while dizygotic (DZ) twin share only 50% (Zheng et al., 2013). Twins are particularly valuable for genetic studies due to their sharing of intrauterine and rearing environments, as well as the genetic similarity and dissimilarity (Tan et al., 2015). By applying structure equation model using MZ and DZ twin samples, the classical twin design is able to estimate heritability for an given phenotype (Duan et al., 2011; Wu et al., 2015). It is usually believed that using unrelated individuals in the GWAS of a quantitative phenotype is better than using related family members because related individuals could be “over-matched” for genotypes. However, researchers reported that for GWAS on a quantitative phenotype with related individuals, little power was lost whilst there are manifold additional advantages, including better quality control, more robust population stratification and fewer false positive (Visscher et al., 2008; Benyamin et al., 2009; Rosenthal et al., 2015).

Here we presented a bivariate twin study design to: (1) assess the genetic correlation between BMI and WHR; (2) determine the BMI-WHR jointly associated genetic variants; and (3) explore the obesity-related pathways in Northern Han Chinese.

## Subjects and methods

### Study population

The study participants were recruited from the Qingdao Twin Registry, China (Pang et al., 2006; Duan et al., 2013). All of the twins in the current study were taken from the latest wave of genetic epidemiology survey on aging phenotypes conducted from 2012 to 2013. Information was collected through questionnaire, extraction of blood, together with anthropometric and laboratory measurements by well-trained clinicians. Zygosity was identified by DNA markers at the laboratory in Qingdao Blood Center. Subjects were included with the following criteria: aged ≥30 years old; Han Chinese; ancestral home is in Shandong Province; free of heart failure, kidney failure, cancer, or severe mental disorder; not pregnant, breast feeding or taking weight-reducing medication within 1 month. A total of 242 pairs of MZ twins including 1 pair of triplet and 140 DZ twins have met the criteria and were included in the current sample. Signed consent forms were obtained from all participants and the study was approved by the Qingdao Center for Disease Control and Prevention Ethics Committee.

### Phenotypes

Weight and standing height were measured for these subjects with lightweight clothes on and shoes removed. Waist circumference was measured at the midpoint between the iliac crest and the lower rib, and hip circumference was measured at the widest circumference over the gluteal muscles. BMI was defined as weight (kilogram) divided by height (meter) squared. WHR was defined as waist circumference (centimeter) divided by hip circumference (centimeter).

### Heritability calculation

A bivariate heritability analysis in 242 MZ and 140 DZ twins was conducted by using the structure equation modeling package Mx (Wu et al., 2011). The Cholesky decomposition model was fitted, with age and sex adjusted. Variations for both phenotype as well as their covariance were decomposed into sources of additive genetic (A), common (shared) environmental (C), and unique (non-shared) environmental (E) parameters. After fitting the full ACE model, the nested models were constructed by dropping the C (AE model) or the A (CE model). The likelihood ratio chi-square tests (-2LL; significant tests indicate significant deterioration in fit) were applied to compare the performances between the full model and its nested models. The index of Akaike's Information Criterion (AIC; parsimony fit index, with lower values indicating the more suitable model) was calculated to estimate the parsimony of the model.

### Genotyping and GWAS

Genomic DNA was extracted from the whole peripheral blood of the subject using QIAamp DNA Blood Mini Kit (Qiagen, GmbH, Hilden, Germany). DNA quantification and integrity were determined by the Nanodrop spectrophotometer (Thermo Fisher Scientific Inc., Wilmington, DE USA) and the 1% agarose electrophoresis, respectively. Genotyping of 278 eligible DNA samples from 139 DZ twin pairs was performed at the BioMiao Biological Technology Company Limited (Beijing, China) using Infinium Omni2.5Exome-8v1.2 genotyping BeadChips (Illumina Inc, San Diego, USA), which covers 2,608,742 SNPs. Only the data from the autosomes were analyzed. The raw data were compiled and tested using GenomeStudio (Illumina) and PLINK tool. All samples have SNP call rates over 95%. Single SNP had to meet the following criteria: minor allele frequencies (MAF) > 0.01, Hardy-Weinberg equilibrium (HWE) significance > 1 × 10^−4^. Finally, 1,365,181 SNPs from 278 samples (139 pairs) were used for the current GWA analysis.

Bivariate GWA analysis of BMI-WHR for 139 DZ twin pairs was done using the program of genome-wide efficient mixed-model analysis (GEMMA) (Zhou and Stephens, 2012). Genetic relatedness matrix and Bayesian sparse linear mixed model were used with age and sex adjusted as covariates. Since our sample size was limited, we did not anticipate genome-wide significant findings (*P* < 0.05 × 10^−8^), and a suggestive evidence level of *P* < 1 × 10^−5^ was adopted (Ran et al., 2013; Loukola et al., 2014). Linkage disequilibrium (LD) block analysis was performed for the jointly significant SNPs located near the same gene by using Haploview system in the “Han Chinese in Beijing, China (CHB)” sample.

### Gene-based analysis

Individual SNP results from the bivariate GWAS were aggregated to conduct a gene-based analysis by using the SNP-set association test implemented in Versatile Gene-based Association Study-2 (VEGAS2, Version of HPC Linux). “1000G East ASIAN” sample was selected. We set *P* < 2.82 × 10^−6^ (0.05/17,723) as genome-wide significant using Bonferroni correction. The *P* < 0.05 was considered as nominal significance level (Xu et al., 2017).

### Gene set enrichment analysis (GSEA)

Significant genes yielded by gene-based analysis were evaluated for enrichment of gene-sets in Reactome and KEGG pathways using GSEA (http://software.broadinstitute.org/gsea/index.jsp). False discovery rate (FDR), analog of hypergeometric *P*-value after correction for multiple hypothesis testing according to Benjamini and Hochberg was calculated to obtain the significant (FDR < 0.05) biological pathways.

### Expression quantitative trait loci (eQTL) analysis

An eQTL search was conducted by submitting the list of suggestively significant SNPs generated by GWAS to HaploReg V4.1 (http://archive.broadinstitute.org/mammals/haploreg/haploreg.php). The GTEx project portal (https://www.gtexportal.org/home/) (as of February 2018) (Consortium, 2013; Loo et al., 2017) was used to identify cis-eQTLs in normal adipose-subcutaneous (*n* = 385), adipose-visceral (omentum) (*n* = 313) or muscle-skeletal (*n* = 491) for our top 20 genes from VEGAS2. Significant level was defined as FDR < 0.05.

## Results

### Heritability analysis

**Figure 1:**
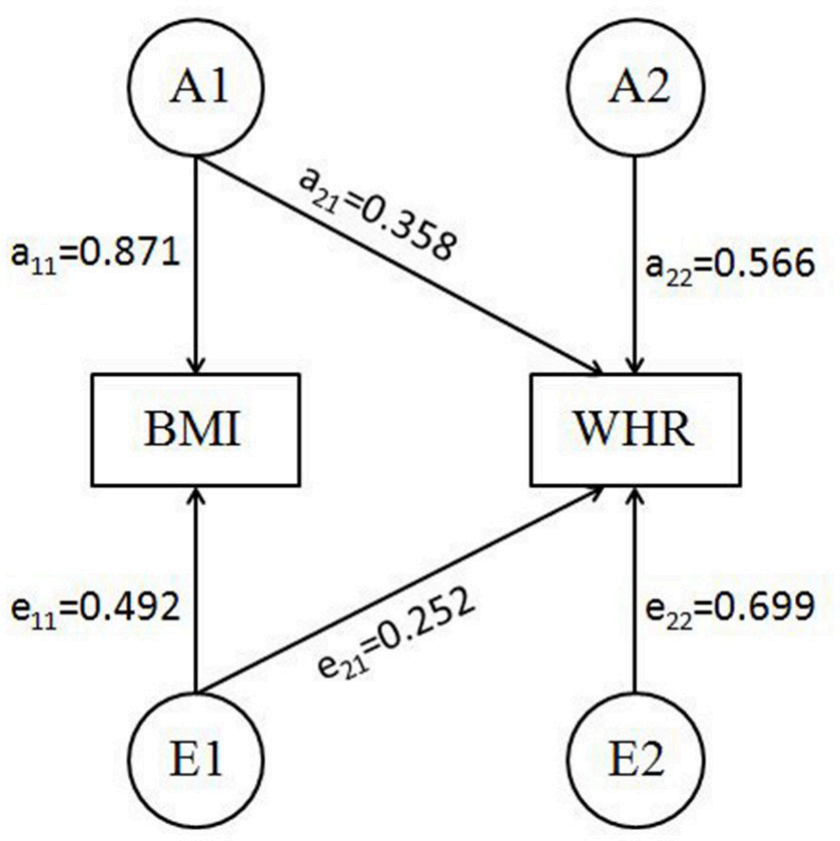
Best bivariate model (AE model) for BMI-WHR with standardized path coefficients (SEM pathway plot). A1, A2 = genetic variance components; E1, E2 = unique environmental variance components; a_11_ through a_22_ = genetic path coefficients of which a_22_ represents specific genetic influence on WHR; e_11_ through e_22_ = unique environmental path coefficients, of which e_22_ represents specific unique environmental influence on WHR.

The basic characteristics of the current sample were summarized in Table 1. The phenotypic correlation between BMI and WHR was 0.39 (*P* < 0.001) and further best fitting Cholesky decomposition model (AE) identified that the genetic correlation between the two phenotypes was 0.53 (95%CI: 0.42–0.64). The standardized path coefficients from best fitting Cholesky decomposition model were presented in Figure 1.

**Table 1:** Descriptive statistics by sex.

**Variable**	**Male**		**Female**		**ALL**	
	***n***	**Mean ±*SD***	***n***	**Mean ±*SD***	***n***	**Mean ±*SD***
Age (y)	372	52.29 ± 8.64	393	50.94 ± 6.67	765	51.6 ± 7.72
Height (cm)	372	165.72 ± 6.56	393	157.76 ± 5.52	765	161.63 ± 7.24
Weight (kg)	372	67.11 ± 11.04	393	60.5 ± 8.82	765	63.72 ± 10.49
BMI (kg/m^2^)	372	24.35 ± 3.07	393	24.31 ± 3.42	765	24.33 ± 3.25
Waist circumference (cm)	372	88.08 ± 9.90	392	81.69 ± 9.81	765	84.79 ± 10.34
Hip circumference (cm)	372	96.77 ± 7.15	392	95.5 ± 7.69	765	96.12 ± 7.47
WHR	372	0.91 ± 0.07	392	0.85 ± 0.06	765	0.88 ± 0.07

### Bivariate GWA analysis

**Figure 2:**
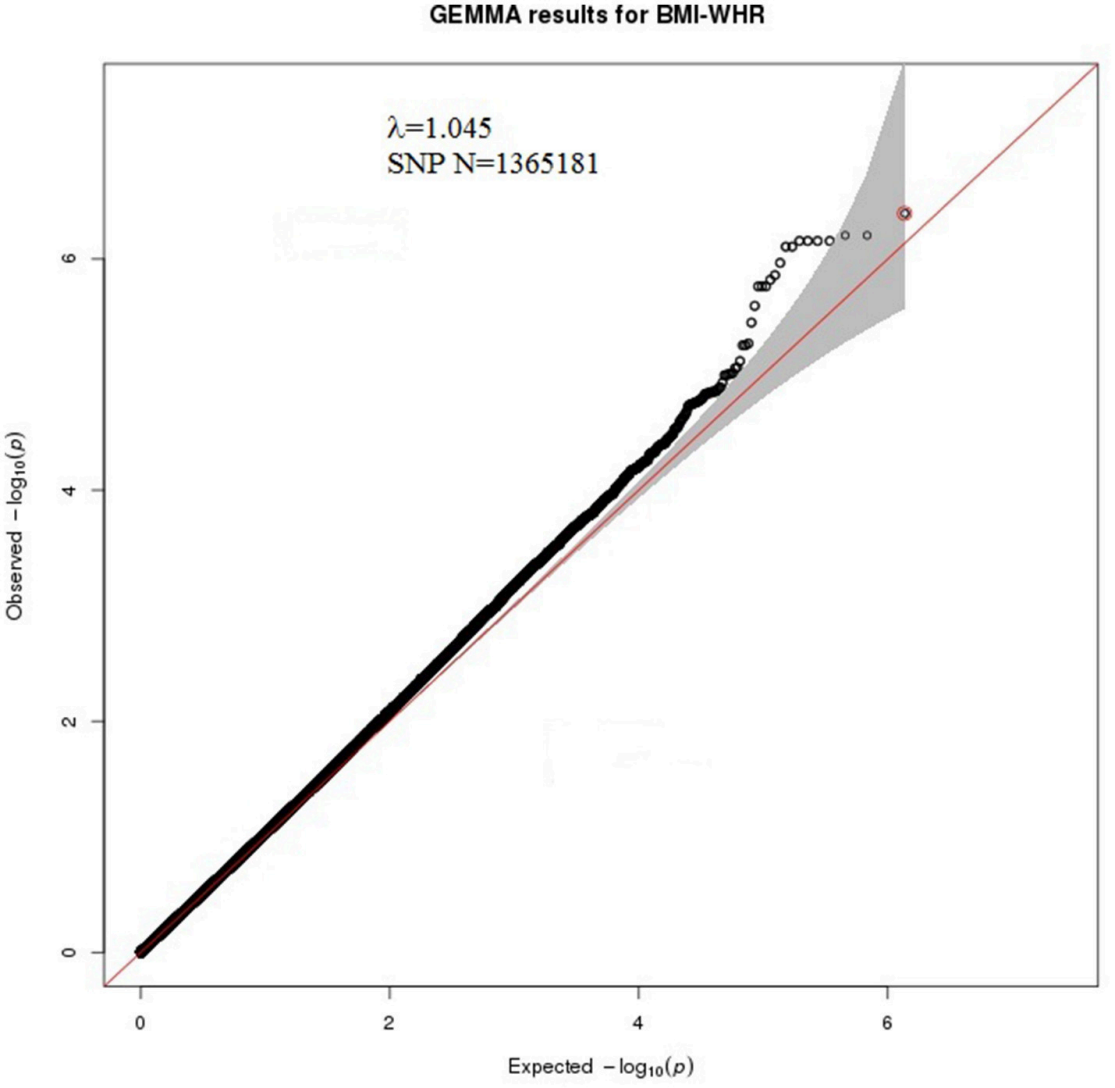
Quantile-quantile plot of χ^2^ test statistics for 1,365,181 SNPs from the bivariate GWA analysis of BMI-WHR, genomic inflation λ = 1.045. The x-axis indicates the expected-log_10_ *P*-values under the null hypothesis. The y-axis shows the observed-log_10_ *P*-values calculated by a bivariate linear mixed model. The red line represents y = x, which corresponds to the null hypothesis of no association. The red dot is the SNP with lowest *P*-value (rs2025924). The gray shaded area shows 95% confidence intervals of the null hypothesis.

**Figure 3:**
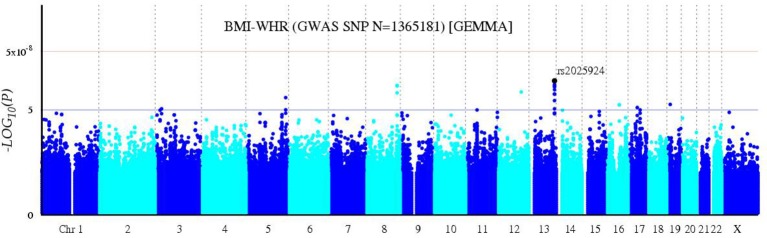
Bivariate Manhattan plot of BMI-WHR. The x-axis represents chromosomal positions and the y-axis represents –log10 *P*-values calculated by a bivariate linear mixed model. The blue and red horizontal lines indicate the suggestive (*P* = 1 × 10^−5^) and genome-wide (*P* = 5 × 10^−8^) significance levels, respectively.

A bivariate GWA analysis on BMI-WHR in 139 DZ twin pairs was performed. The relationship between the observed and expected GWAS *P*-values was illustrated in the quantile-quantile plot (Figure 2). There was a slight deviation in the upper right tail from the null hypothesis, suggesting an evidence for weak association. A low level of λ-statistic (λ = 1.045) indicated no evidence of genomic inflation or bias from population stratification. As illustrated from the Manhattan plot (Figure 3), although none of the SNPs reached the genome-wide significance level, 26 SNPs involved in 10 genes that were suggestively associated with BMI-WHR (*P* < 10^−5^) were identified. The characteristics of the 26 SNPs were summarized in Table 2. Considering that several SNPs were mapped near the same gene with the similar beta value, LD block analyses were performed and identified that 11 SNPs near the *LINC0034* gene (from *r*^2^ = 0.801 for rs7320405 and rs9586994 to *r*^2^ = 1 for rs2025924 and rs13378734), 4 SNPs near the *LOC105375763* gene (*r*^2^ = 1 for each other), 2 SNPs near the *DOCK2* gene (*r*^2^ = 1), 2 SNPs near the *LOC101928945* gene (*r*^2^ = 1) were in the same block (the conventional threshold of *r*^2^ > 0.8). The SNP rs201664108 near the *LOC105375763* gene was not available in Haploview. In addition, we mapped the genome-wide significant SNPs reported from previous meta-analyses, including 97 BMI loci (Locke et al., 2015), 49 WHR adjusted for BMI loci (Shungin et al., 2015) and 10 BMI loci in East Asian (Wen et al., 2012), to the region of 100 kilo-bases (kb) upstream and downstream from the 26 SNPs identified by our study. No overlap was found.

**Table 2:** SNPs that reached *p* < 10^−5^ from bivariate GWA analysis of BMI-WHR (NCBI build 37).

**SNP**	**Chr**.	**Position**	**β-value**	***S.E*.**	***P*-value**	**Nearest GENE**
rs2025924	13	106446477	0.80	0.34	4.04E-07	*LINC00343*
rs78826453	13	106451587	0.79	0.33	6.26E-07	*LINC00343*
rs80230511	13	106466262	0.79	0.33	6.26E-07	*LINC00343*
rs715969	8	132473863	0.15	0.60	6.98E-07	*LOC105375763*
rs1507456	8	132476129	0.15	0.60	6.98E-07	*LOC105375763*
rs1118349	8	132502614	0.15	0.60	6.98E-07	*LOC105375763*
rs60065489	8	132506650	0.15	0.60	6.98E-07	*LOC105375763*
rs729426	13	106480081	0.77	0.29	7.83E-07	*LINC00343*
rs13378734	13	106485240	0.77	0.29	7.83E-07	*LINC00343*
rs7335212	13	106476076	0.74	0.24	1.08E-06	*LINC00343*
rs12227147	12	99243021	−0.31	−0.65	1.38E-06	*ANKS1B*
rs201664108	8	132491815	0.15	0.59	1.52E-06	*LOC105375763*
rs72663838	13	106487254	0.77	0.29	1.73E-06	*LINC00343*
rs59412652	13	106488121	0.77	0.29	1.73E-06	*LINC00343*
rs9586994	13	106488608	0.77	0.29	1.73E-06	*LINC00343*
rs35316183	5	169122177	−0.01	0.46	2.55E-06	*DOCK2*
rs7987002	13	106447755	0.76	0.35	3.55E-06	*LINC00343*
rs79817709	19	10596872	−0.84	−0.01	5.39E-06	*KEAP1*
rs61554960	16	51278533	0.02	−0.37	5.59E-06	*LOC101928945*
rs7500931	16	51279824	0.02	−0.37	5.59E-06	*LOC101928945*
rs35704505	17	35072498	−0.39	0.98	7.64E-06	*LOC105371750*
rs76924951	3	22803519	−0.04	0.48	8.74E-06	*LOC100421669*
rs7320405	13	106482474	0.68	0.20	8.80E-06	*LINC00343*
rs2112705	5	169124478	−0.06	0.40	9.69E-06	*DOCK2*
rs2242044	11	45388622	−0.15	−0.39	9.91E-06	*LOC399886*
rs617182	17	47307274	−0.41	−0.29	9.92E-06	*LOC105371816*

### Gene-based analysis

Results of gene-based analysis using VEGAS2 were reported in Supplementary Table 1. While no gene achieved genome-wide significance, a total of 291 genes were found to be nominally associated with BMI-WHR (*P* < 0.05), and we reported the top 20 genes ranked by their *P*-value in Table 3.

**Table 3:** Top 20 genes from gene-based results based on bivariate GWA analysis of BMI-WHR.

**Chr**.	**Gene**	**snpN**	**From**	**To**	***P***
5q35.3	*SLC34A1*	29	176744040	176758455	3.60E-05
5q35.3	*F12*	28	176761744	176769183	3.80E-05
5q35.3	*RGS14*	28	176717449	176732205	4.20E-05
5q35.3	*PFN3*	26	176759713	176760243	4.30E-05
17q11.2	*LOC116236*	27	24912436	24918174	6.80E-05
17q11.2	*TP53I13*	28	24919864	24924301	7.00E-05
4p15.32	*MED28*	28	17225370	17235258	8.40E-05
4p15.32	*LAP3*	26	17188024	17218688	1.40E-04
17q11.2	*GNGT2*	37	44638595	44641742	1.70E-04
17q11.2	*GIT1*	33	24924612	24940736	1.80E-04
11q24.3-q25	*SNX19*	71	130250975	130291592	1.90E-04
17q21.32	*PHOSPHO1*	33	44655730	44663127	2.10E-04
22q12.1	*ASPHD2*	69	25155279	25170978	2.30E-04
17q11.2	*CORO6*	26	24965899	24972620	2.50E-04
17q11.2	*ANKRD13B*	34	24944652	24965905	2.80E-04
17q21.32	*ABI3*	39	44642587	44655586	2.80E-04
11p11.2	*SYT13*	63	45218428	45264460	2.80E-04
17q21.32	*B4GALNT2*	49	44565327	44602121	2.90E-04
1p35.2	*COL16A1*	61	31890434	31942355	3.30E-04
1p35.2	*HCRTR1*	40	31857227	31865301	3.30E-04

### GSEA

Gene set enrichment analysis with 291 significant genes using GSEA reported 6 biological pathways (Table 4). In these 6 pathways, there were 5 REACTOME pathways which are involved in hemostasis, G protein-coupled receptor (GPCR) signaling, platelet production, GPCR ligand binding, and gastrin-CREB signaling. The only KEGG pathway was the olfactory transduction pathway.

**Table 4:** The pathways with FDR < 0.05 discovered by GSEA.

**Gene-set name [# genes (K)]**	**Description**	**Significant genes by VEGAS2 in overlapping gene set**	***P*-value**	**FDR**
**REACTOME PATHWAYS**				
HEMOSTASIS [466]	Genes involved in Hemostasis	*GNGT2, DGKB, KIF4B, KIF15, DOCK2, WEE1, DOCK6, DOCK4, MMP1, NOS2, ACTN1, F12, ANGPT2*	7.47E-6	6.42E-3
SIGNALING_BY_GPCR [920]	Genes involved in Signaling by GPCR	*GNGT2, DGKB, OR52K2, OR4D1, OR52K1, OR2L8, OR2AK2, TRHR, HCRTR1, P2RY2, CCK, GLP1R, HRH2, GPR55, FZD2, EMR3, MMP3*	6.43E-5	1.84E-2
FACTORS_INVOLVED_IN_MEGAKARYOCYTE_DEVELOPMENT_AND_PLATELET_PRODUCTION [132]	Genes involved in Factors involved in megakaryocyte development and platelet production	*KIF4B, KIF15, DOCK2, WEE1, DOCK6, DOCK4*	1.74E-4	3.73E-2
GPCR_LIGAND_BINDING [408]	Genes involved in GPCR ligand binding	*GNGT2, TRHR, HCRTR1, P2RY2, CCK, GLP1R, HRH2, GPR55, FZD2, EMR3*	2.40E-4	4.13E-2
GASTRIN_CREB_SIGNALLING_PATHWAY_VIA_PKC_AND_MAPK [205]	Genes involved in Gastrin-CREB signaling pathway via PKC and MAPK	*GNGT2, DGKB, TRHR, HCRTR1, P2RY2, CCK, MMP3*	2.95E-4	4.22E-2
**KEGG PATHWAYS**				
OLFACTORY_TRANSDUCTION [389]	Olfactory transduction	*OR52K2, OR4D1, OR52K1, OR2L8, OR2AK2, CALML5, CALML3, CLCA1, CNGB1, CLCA4, CLCA2*	3.32E-5	1.43E-2

*CREB, cyclic adenosine 3′,5′-monophosphate (cAMP) response element binding. GPCR, G Protein-Coupled Receptor*.

### eQTL analysis

HaploReg presented all of the SNPs that are in LD with our 26 queried SNPs. Among them, rs2242044 from our GWAS and 2 other variants (rs1868910 and rs10838458) with *r*^2^ > 0.8 were identified as significant eQTLs in both the adipose-subcutaneous and adipose-visceral tissues. GTEx Portal showed that *P*-values of rs2242044 were 1.7 × 10^−9^ and 4.4 × 10^−15^ for normal adipose-subcutaneous and adipose-visceral tissue, respectively. We also queried our top 20 genes from VEGAS2 using the GTEx Portal, and it identified that 13 of them harbored statistically significant cis-eQTLs in at least one relevant tissue (Supplementary Table 2).

## Discussion

This twin study documented four major findings. First, the Cholesky heritability model in 242 MZ and 140 DZ twin pairs suggested an important genetic component in the phenotypic correlation between BMI and WHR. Second, bivariate GWAS in 139 pairs of DZ twins and further gene-based analysis revealed several associated SNPs and gene-based loci supported by some previous reports. Third, GSEA identified pathways involved in hemostasis, platelet production, GPCR signaling, Gastrin-CREB signaling and aolfactory transduction, all of which were associated with BMI-WHR. Fourth, after querying suggestively significant SNPs, or SNPs in high LD with them by using HaploReg and GTEx Portal, we determined that rs2242044 was a significant cis-eQTL in both the normal adipose-subcutaneous and adipose-visceral tissue.

Our estimate of a moderate genetic correlation between BMI and WHR was similar to previous works in other populations (Li et al., 2006; Zabaneh et al., 2009) and it confirmed the necessity for further bivariate GWA analysis for discovering relevant pleiotropic gene variants. The current GWAS identified 26 SNPs with genome-wide-suggestive *P*-value cutoff 1 × 10^−5^ (Table 2). Most of them were near to the RNA genes that not yet characterized and/or their neighboring protein-coding genes that have not been previously identified to be associated with obesity. However, a connection between rs79817709 and obesity is biologically plausible. SNP rs79817709 lies in 3′UTR (utr variant 3 prime) of *KEAP1* gene. KEAP1 protein is an inhibitor of nuclear factor (erythroid-derived 2)-like 2 (Nrf2) by blocking Nrf2 translocation to the nucleus and also promoting its degradation, and lack of Nrf2 can ameliorate insulin resistance, adipogenesis and adipocyte differentiation (Pi et al., 2010; Zhang et al., 2015).

The gene-based analysis focused on several associated chromosome regions, including 5q35.3, 17q11.2, 4p15.32, 1p35.2 and so on (Table 3). Our findings were supported by previous reports on the linkage of chromosome 5q35 to obesity-related phenotypes in Caucasians population (Zhao et al., 2007; He et al., 2008), the linkage of 1p35.2 to plasma adiponectin concentrations in Genetics of Lipid Lowering Drugs and Diet Network Study (Rasmussen-Torvik et al., 2009), as well as the linkage of 4p15.3 to hip circumference in post-menopausal women from a United States mid-western population (Kelemen et al., 2010). Although there was no direct evidence that the chromosome 17q11.2 region was linked to obesity traits, a case report should be noted that a 3-year-old girl with a microduplication at 17q11.2 have developed obesity within the first 6 months of life (White et al., 2013).

*F12*, our second top gene from VEGAS2 results (Table 3), encodes coagulation factor XII which circulates in blood as a zymogen. It has been showed that the activity of coagulation factor XII increased in the obese individual and has strong correlation with the measures of adiposity and insulin resistance in a number of observational studies (Bowles et al., 2003; Kotronen et al., 2011). The twelfth top gene, *PHOSPHO1*, is involved in the pathways of metabolism and glycerophospholipid biosynthesis. A previous study found that DNA methylation at the *PHOSPHO1* was associated with type 2 diabetes (Chambers et al., 2015) and positively correlated with high density lipoprotein levels (Dayeh et al., 2016). *HCRTR1*, the twentieth top gene, encodes hypocretin receptor type 1, a protein belonging to the G-protein coupled receptor family. This protein binds with neuropeptide orexin A, and orexin neurons provide a critical link between peripheral energy balance and the central mechanisms that coordinate sleep/wakefulness and motivate behaviors such as food seeking (Kaewsutthi et al., 2016).

The results of GSEA indicated that 34 (Table 4) of the 291 significant genes (Supplementary Table 1) overlapped with 6 biological pathways. Apart from the aforementioned *F12* and *HCRTR1* genes, several other genes should be also noted, i.e., *DOCK2, DOCK6, DGKB, GLP1R, TRHR, MMP1, GPR55, CCK*, and *OR2AK2*. *DOCK2* was the only gene identified both by our GWAS and GSEA. Dedicator of cytokinesis 2 (DOCK2) is involved in several inflammatory diseases. A recent experimental study indicated that DOCK2 deficiency might protect mice from high-fat-diet induced obesity by reducing adipose tissue inflammation and increasing energy expenditure (Guo et al., 2017). Our findings on the role of *DOCK6* and *DGKB* in BMI-WHR were supported by two meta-analyses respectively which identified genome-wide significant signals near *DOCK6* for total cholesterol in Hispanics (Below et al., 2016) and near DGKB for beta cell function in East Asians (Hong et al., 2014).

*GLP1R* gene encodes a 7-transmembrane protein that functions as a receptor for glucagon-like peptide 1 hormone, which stimulates glucose-induced insulin secretion. *TRHR* gene encodes the thyrotropin-releasing hormone receptor and regulates crucial biological functions, including the increase of basal cell metabolism, growth, thermogenesis, total energy expenditure, lipid and carbohydrate catabolism, as well as myogenesis in skeletal muscle (Costa-Urrutia et al., 2017). *MMP1* gene encodes a member of the peptidase M10 family of matrix metalloproteinases which are involved in the breakdown of extracellular matrix in normal physiological processes. Previous candidate gene studies on obesity and its related traits have reported *GLP1R* in European Americans (Li et al., 2014), *TRHR* in Mexican-Mestizos (Costa-Urrutia et al., 2017) and *MMP1* in Koreans (Nho et al., 2008). Our results further consolidated these associations in Northern Han Chinese.

Our observations on the significant associations of *GPR55* and *CCK* with BMI-WHR were also supported by two interesting experimental studies: Compared to wild-type mice, *GPR55*-null mice exhibited significantly increased fat-mass and insulin resistance as well as decreased spontaneous locomotor activity and physical activity (Meadows et al., 2016); *CCK* knockout mice were resistant to high-fat diet-induced obesity (Lo et al., 2010). It suggested that these two genes might play important roles in human energy balance and metabolism. Besides, *OR2AK2*, a member of olfactory receptor genes family which located in chromosome 1q, involved in both GPCR signaling pathway and olfactory signaling pathway. It has been found that the predicted damaging missense variants in olfactory receptor genes on chromosome 1q and rare damaging variants in protocadherin beta-cluster genes on chromosome 5q31 co-localized in subjects with extreme obesity (Mariman et al., 2015). The functions of other genes in terms of obesity were unknown, whereas they may be interesting potential candidates for future research.

Our eQTL analysis showed that rs2242044 was a significant cis-eQTL in both the normal adipose-subcutaneous and adipose-visceral tissue. LOC399886, which is the closest gene to rs2242044, is an uncharacterized ncRNA. Whether or not rs2242044, as an eQTL, controls the expression of LOC399886 in regulating obesity requires further investigation.

We utilized a sample of adult Chinese twins with a homogenous genetic background to conduct a bivariate GWA analysis on BMI-WHR. Comparing with traditional univariate GWAS in unrelated population, this design has a stronger power in identifying genes with pleiotropic effect and may help reveal the interconnected pathophysiological networks for a spectrum of common human diseases such as obesity (Ran et al., 2013; Loukola et al., 2014). However, there are some potential limitations in our study. Firstly, in many cases, the loci identified by our GWAS harbor few, if any, annotated genes with clear connections to the biology of obesity. Our findings presented here still need to be further validated. Secondly, we couldn't replicate our bivariate GWAS results when compared with previously implicated meta-analyses with large sample sizes (Wen et al., 2012; Locke et al., 2015; Shungin et al., 2015), perhaps due to ethnic differences in genetic background and sample characteristics. Thirdly, although 13 of the top 20 genes harbored significant cis-eQTLs in adipose or muscle-skeletal tissue, it is not clear that whether the SNPs underlying the top enriched genes as well as their LD SNPs overlap with these cis-eQTLs.

In conclusion, this twin study identified several BMI-WHR jointly associated genetic loci, genomic regions and pathways that are biologically meaningful. It suggested that there are still many biological mechanisms related to obesity waiting to be discovered, and applying methods in this article on larger dataset may yields more genes and pathways that are biological meaningful.

## Author notes

The SNP data has been deposited in the European Variation Archive (EVA), (accession no. PRJEB23749).

## Author contributions

YW, ZP, DZ, and QT designed the study. HD, CX, XT, and ZP collected the data. YW, WW, WJ, and QT analyzed the data and interpreted the results. YW wrote the first version of the article. All authors reviewed and edited the manuscript and approved the final version.

### Conflict of interest statement

The authors declare that the research was conducted in the absence of any commercial or financial relationships that could be construed as a potential conflict of interest. The reviewer LS and handling Editor declared their shared affiliation.
