# Supplementary material for: Genetics of Obesity Traits: A Bivariate Genome-Wide Association Analysis
Source: Front Genet. 2018 May 16;9:179. doi: 10.3389/fgene.2018.00179 (PMC5964872; doi:10.3389/fgene.2018.00179)
Supplement: Supplementary file 1 [file Table_1.docx]

**Supplementary Table 1**

**Genes that reached p<0.05 from gene-based results based on bivariate GWA analysis of BMI-WHR**

| Chr. | gene | snpN | from | to | P |
| --- | --- | --- | --- | --- | --- |
| 5 | SLC34A1 | 29 | 176744040 | 176758455 | 3.60E-05 |
| 5 | F12 | 28 | 176761744 | 176769183 | 3.80E-05 |
| 5 | RGS14 | 28 | 176717449 | 176732205 | 4.20E-05 |
| 5 | PFN3 | 26 | 176759713 | 176760243 | 4.30E-05 |
| 17 | LOC116236 | 27 | 24912436 | 24918174 | 6.80E-05 |
| 17 | TP53I13 | 28 | 24919864 | 24924301 | 7.00E-05 |
| 4 | MED28 | 28 | 17225370 | 17235258 | 8.40E-05 |
| 4 | LAP3 | 26 | 17188024 | 17218688 | 1.40E-04 |
| 17 | GNGT2 | 37 | 44638595 | 44641742 | 1.70E-04 |
| 17 | GIT1 | 33 | 24924612 | 24940736 | 1.80E-04 |
| 11 | SNX19 | 71 | 130250975 | 130291592 | 1.90E-04 |
| 17 | PHOSPHO1 | 33 | 44655730 | 44663127 | 2.10E-04 |
| 22 | ASPHD2 | 69 | 25155279 | 25170978 | 2.30E-04 |
| 17 | CORO6 | 26 | 24965899 | 24972620 | 2.50E-04 |
| 17 | ANKRD13B | 34 | 24944652 | 24965905 | 2.80E-04 |
| 17 | ABI3 | 39 | 44642587 | 44655586 | 2.80E-04 |
| 11 | SYT13 | 63 | 45218428 | 45264460 | 2.80E-04 |
| 17 | B4GALNT2 | 49 | 44565327 | 44602121 | 2.90E-04 |
| 1 | COL16A1 | 61 | 31890434 | 31942355 | 3.30E-04 |
| 1 | HCRTR1 | 40 | 31857227 | 31865301 | 3.30E-04 |
| 1 | PEF1 | 43 | 31868057 | 31883064 | 3.40E-04 |
| 5 | GRK6 | 24 | 176786292 | 176802456 | 4.10E-04 |
| 17 | IKZF3 | 31 | 35174724 | 35273967 | 4.10E-04 |
| 11 | PRDM11 | 84 | 45072139 | 45203479 | 4.10E-04 |
| 11 | C11orf67 | 24 | 77209855 | 77261046 | 4.70E-04 |
| 22 | HPS4 | 80 | 25177445 | 25209820 | 5.20E-04 |
| 1 | ZBTB8 | 23 | 32777358 | 32844129 | 5.70E-04 |
| 8 | DENND3 | 87 | 142207901 | 142275082 | 5.90E-04 |
| 1 | TINAGL1 | 38 | 31814746 | 31825874 | 6.00E-04 |
| 17 | ZPBP2 | 30 | 35277980 | 35287675 | 6.10E-04 |
| 22 | TFIP11 | 78 | 25217894 | 25238437 | 6.30E-04 |
| 1 | TINAGL1 | 38 | 31814746 | 31825874 | 6.40E-04 |
| 22 | SRRD | 68 | 25209849 | 25217904 | 6.90E-04 |
| 22 | SRRD | 68 | 25209849 | 25217904 | 7.30E-04 |
| 9 | ANGPTL2 | 13 | 128889448 | 128924865 | 7.40E-04 |
| 1 | FOXD3 | 31 | 63561317 | 63563385 | 7.50E-04 |
| 15 | WHDC1 | 37 | 81275026 | 81300667 | 7.70E-04 |
| 1 | FOXD3 | 31 | 63561317 | 63563385 | 7.90E-04 |
| 22 | RFPL3 | 58 | 31080871 | 31087148 | 8.20E-04 |
| 22 | RFPL3 | 58 | 31080871 | 31087148 | 8.50E-04 |
| 11 | RSF1 | 40 | 77054921 | 77209528 | 8.50E-04 |
| 4 | CXXC4 | 35 | 105612791 | 105631916 | 1.00E-03 |
| 2 | GPR55 | 62 | 231480286 | 231498185 | 1.00E-03 |
| 15 | HOMER2 | 43 | 81314789 | 81412477 | 1.10E-03 |
| 16 | ACSM1 | 34 | 20542059 | 20610079 | 1.10E-03 |
| 5 | PAPD4 | 81 | 78943998 | 79018227 | 1.20E-03 |
| 2 | PCBP1 | 14 | 70168204 | 70169836 | 1.20E-03 |
| 17 | GSDML | 36 | 35314373 | 35328429 | 1.20E-03 |
| 11 | CLNS1A | 35 | 77004846 | 77026495 | 1.20E-03 |
| 13 | UGCGL2 | 61 | 95251836 | 95503638 | 1.30E-03 |
| 17 | GPATCH8 | 29 | 39828175 | 39936328 | 1.30E-03 |
| 19 | CCDC130 | 28 | 13719752 | 13735106 | 1.40E-03 |
| 7 | DOCK4 | 214 | 111153399 | 111633698 | 1.40E-03 |
| 11 | INTS4 | 52 | 77267413 | 77383365 | 1.40E-03 |
| 10 | OLAH | 52 | 15125950 | 15155857 | 1.40E-03 |
| 4 | SPARCL1 | 72 | 88613511 | 88669679 | 1.50E-03 |
| 19 | MGC3207 | 28 | 13736336 | 13746096 | 1.50E-03 |
| 14 | RNASE3 | 32 | 20429401 | 20430347 | 1.50E-03 |
| 22 | C22orf28 | 88 | 31113561 | 31138274 | 1.60E-03 |
| 5 | ANKRD34B | 25 | 79888329 | 79901854 | 1.60E-03 |
| 16 | ZNF319 | 43 | 56586073 | 56591263 | 1.60E-03 |
| 5 | PRR7 | 24 | 176806401 | 176815889 | 1.70E-03 |
| 2 | ITM2C | 62 | 231437864 | 231452207 | 1.70E-03 |
| 6 | GLP1R | 90 | 39124534 | 39163498 | 1.70E-03 |
| 12 | ZNF705A | 27 | 8216416 | 8223909 | 1.70E-03 |
| 11 | AQP11 | 31 | 76978327 | 76998341 | 1.80E-03 |
| 8 | TRHR | 31 | 110168901 | 110200988 | 1.90E-03 |
| 16 | TEPP | 51 | 56567839 | 56579518 | 1.90E-03 |
| 6 | HCG27 | 292 | 31273577 | 31279724 | 1.90E-03 |
| 13 | IPO5 | 48 | 97403929 | 97474551 | 1.90E-03 |
| 16 | C16orf57 | 43 | 56592805 | 56613023 | 2.00E-03 |
| 19 | C19orf53 | 29 | 13746256 | 13750586 | 2.10E-03 |
| 4 | SLC10A7 | 86 | 147395627 | 147662535 | 2.20E-03 |
| 19 | ZSWIM4 | 39 | 13767273 | 13804044 | 2.30E-03 |
| 19 | SPC24 | 61 | 11118830 | 11127484 | 2.30E-03 |
| 9 | JMJD2C | 321 | 6747653 | 7165648 | 2.30E-03 |
| 1 | CLCA4 | 35 | 86785346 | 86819020 | 2.40E-03 |
| 19 | LDLR | 62 | 11061056 | 11105505 | 2.40E-03 |
| 9 | RALGPS1 | 54 | 128716873 | 129025264 | 2.60E-03 |
| 1 | CLCA1 | 87 | 86707113 | 86738562 | 2.70E-03 |
| 19 | SIGLEC14 | 54 | 56837617 | 56841944 | 2.70E-03 |
| 7 | FLJ22374 | 125 | 30777557 | 30898527 | 2.90E-03 |
| 2 | SDPR | 29 | 192407280 | 192420226 | 2.90E-03 |
| 19 | KANK2 | 81 | 11135945 | 11167496 | 2.90E-03 |
| 12 | RIC8B | 32 | 105692528 | 105807224 | 3.10E-03 |
| 4 | NARG1 | 25 | 140442125 | 140531385 | 3.40E-03 |
| 15 | FSD2 | 42 | 81225077 | 81271860 | 3.50E-03 |
| 3 | IQCB1 | 26 | 122971299 | 123036616 | 3.70E-03 |
| 7 | ZNF277 | 56 | 111633878 | 111771225 | 3.80E-03 |
| 7 | INMT | 78 | 30758275 | 30763743 | 3.80E-03 |
| 10 | CALML3 | 62 | 5556923 | 5558225 | 3.80E-03 |
| 6 | C6orf64 | 94 | 39179817 | 39190843 | 3.90E-03 |
| 8 | SLC45A4 | 69 | 142290051 | 142307855 | 4.10E-03 |
| 11 | MMP1 | 63 | 102165860 | 102174104 | 4.20E-03 |
| 16 | MMP15 | 34 | 56616782 | 56638305 | 4.30E-03 |
| 9 | GARNL3 | 62 | 129066966 | 129195649 | 4.30E-03 |
| 3 | YEATS2 | 61 | 184898299 | 185013102 | 4.40E-03 |
| 13 | CRYL1 | 102 | 19875805 | 19998012 | 4.70E-03 |
| 3 | DCBLD2 | 37 | 99997503 | 100103223 | 4.80E-03 |
| 2 | SH3BP4 | 99 | 235525366 | 235629097 | 5.00E-03 |
| 11 | TPCN2 | 77 | 68572925 | 68614648 | 5.30E-03 |
| 4 | NDUFC1 | 23 | 140430540 | 140436407 | 5.50E-03 |
| 3 | TMF1 | 43 | 69151667 | 69184174 | 5.50E-03 |
| 15 | FAM148A | 40 | 60146467 | 60150408 | 5.60E-03 |
| 9 | ASTN2 | 560 | 118227327 | 119217138 | 5.80E-03 |
| 16 | ACSM2B | 26 | 20455583 | 20495196 | 5.80E-03 |
| 11 | FBXO3 | 61 | 33719065 | 33752647 | 5.80E-03 |
| 15 | VPS13C | 124 | 59931881 | 60139939 | 5.90E-03 |
| 6 | PDSS2 | 98 | 107580453 | 107887472 | 6.10E-03 |
| 19 | SIGLEC5 | 61 | 56806995 | 56825530 | 6.40E-03 |
| 6 | EPB41L2 | 112 | 131202180 | 131426015 | 6.40E-03 |
| 3 | UBA3 | 35 | 69186570 | 69212214 | 6.40E-03 |
| 2 | FMNL2 | 173 | 152899996 | 153214594 | 6.40E-03 |
| 17 | FZD2 | 33 | 39990450 | 39992433 | 6.50E-03 |
| 6 | POU5F1 | 393 | 31240092 | 31246430 | 6.50E-03 |
| 4 | FHDC1 | 65 | 154083584 | 154120298 | 6.60E-03 |
| 2 | ASPRV1 | 32 | 70040727 | 70042901 | 6.60E-03 |
| 10 | HPSE2 | 230 | 100208866 | 100985609 | 6.70E-03 |
| 4 | OSAP | 23 | 140406766 | 140420942 | 6.80E-03 |
| 6 | TCF19 | 418 | 31234281 | 31239971 | 6.90E-03 |
| 11 | ZNF143 | 28 | 9439088 | 9506647 | 7.00E-03 |
| 8 | XKR4 | 198 | 56177570 | 56601264 | 7.00E-03 |
| 20 | C20orf107 | 44 | 54541708 | 54544983 | 7.20E-03 |
| 19 | ZNF333 | 57 | 14661869 | 14692772 | 7.40E-03 |
| 5 | FAM151B | 39 | 79819555 | 79873962 | 7.50E-03 |
| 2 | TET3 | 43 | 74126957 | 74188810 | 7.50E-03 |
| 20 | C20orf106 | 43 | 54532954 | 54534605 | 7.50E-03 |
| 15 | KIAA0574 | 222 | 27199746 | 27650219 | 7.70E-03 |
| 3 | TMEM42 | 29 | 44878411 | 44882158 | 7.70E-03 |
| 6 | CCHCR1 | 438 | 31218194 | 31233994 | 7.80E-03 |
| 4 | UBE2K | 41 | 39376058 | 39460805 | 7.80E-03 |
| 11 | WEE1 | 30 | 9550915 | 9567874 | 8.00E-03 |
| 10 | TCTN3 | 36 | 97413147 | 97443890 | 8.00E-03 |
| 3 | TRNT1 | 81 | 3143599 | 3165706 | 8.70E-03 |
| 17 | TAOK1 | 50 | 24742068 | 24895628 | 8.90E-03 |
| 5 | DBN1 | 28 | 176816219 | 176833300 | 9.00E-03 |
| 14 | OTX2 | 48 | 56337177 | 56346937 | 9.00E-03 |
| 6 | C6orf89 | 53 | 36961617 | 37004718 | 9.10E-03 |
| 7 | VWC2 | 47 | 49783802 | 49922684 | 9.60E-03 |
| 6 | UTRN | 200 | 144654565 | 145215863 | 9.80E-03 |
| 10 | ACBD7 | 54 | 15157479 | 15170781 | 1.00E-02 |
| 11 | LTBP3 | 29 | 65062605 | 65082006 | 1.00E-02 |
| 7 | PHTF2 | 58 | 77266044 | 77424757 | 1.00E-02 |
| 1 | MAEL | 45 | 165225142 | 165258071 | 1.00E-02 |
| 11 | KIAA1377 | 42 | 101290955 | 101377003 | 1.10E-02 |
| 3 | GBE1 | 66 | 81621539 | 81893640 | 1.10E-02 |
| 16 | CNGB1 | 99 | 56475205 | 56562513 | 1.10E-02 |
| 12 | CLEC4A | 41 | 8167494 | 8182470 | 1.10E-02 |
| 3 | ST3GAL6 | 39 | 99934261 | 99995926 | 1.10E-02 |
| 11 | MMP3 | 57 | 102211737 | 102219552 | 1.10E-02 |
| 11 | SSSCA1 | 28 | 65094518 | 65095815 | 1.10E-02 |
| 3 | CRBN | 77 | 3166695 | 3196390 | 1.20E-02 |
| 6 | GCLC | 79 | 53470098 | 53517790 | 1.20E-02 |
| 3 | C3orf64 | 39 | 69107057 | 69145464 | 1.20E-02 |
| 17 | SSH2 | 67 | 24977090 | 25281144 | 1.20E-02 |
| 22 | BPIL2 | 110 | 31140289 | 31183373 | 1.30E-02 |
| 19 | KIR3DX1 | 52 | 59735819 | 59748836 | 1.30E-02 |
| 10 | RBM17 | 74 | 6171012 | 6198847 | 1.30E-02 |
| 11 | CHST1 | 48 | 45627002 | 45643748 | 1.30E-02 |
| 5 | MRPL22 | 39 | 154300825 | 154329164 | 1.40E-02 |
| 3 | KIF15 | 38 | 44778212 | 44869752 | 1.40E-02 |
| 1 | C1orf32 | 35 | 165154619 | 165211185 | 1.40E-02 |
| 20 | GCNT7 | 58 | 54499954 | 54534613 | 1.50E-02 |
| 6 | PSORS1C2 | 413 | 31213289 | 31215106 | 1.50E-02 |
| 4 | CCRN4L | 26 | 140156392 | 140186543 | 1.50E-02 |
| 11 | P2RY2 | 48 | 72606991 | 72625043 | 1.50E-02 |
| 17 | CD300E | 73 | 70120306 | 70131396 | 1.50E-02 |
| 13 | PABPC3 | 63 | 24568275 | 24570704 | 1.50E-02 |
| 20 | C20orf43 | 58 | 54477053 | 54527349 | 1.50E-02 |
| 11 | OR52K2 | 63 | 4427145 | 4428090 | 1.50E-02 |
| 6 | OLIG3 | 40 | 137855028 | 137857224 | 1.60E-02 |
| 3 | IL5RA | 120 | 3086420 | 3127031 | 1.60E-02 |
| 1 | PTPN14 | 120 | 212597633 | 212791265 | 1.60E-02 |
| 10 | CALML5 | 55 | 5530659 | 5531510 | 1.60E-02 |
| 7 | DGKB | 397 | 14151198 | 14847600 | 1.60E-02 |
| 20 | LPIN3 | 39 | 39402973 | 39422636 | 1.60E-02 |
| 4 | DDX60L | 107 | 169514460 | 169638213 | 1.60E-02 |
| 17 | OR4D1 | 36 | 53587513 | 53588446 | 1.70E-02 |
| 12 | FLJ22662 | 37 | 14547863 | 14612058 | 1.70E-02 |
| 11 | FAM89B | 32 | 65096395 | 65098245 | 1.70E-02 |
| 3 | GPR156 | 33 | 121368568 | 121445635 | 1.80E-02 |
| 19 | DOCK6 | 78 | 11170970 | 11234157 | 1.80E-02 |
| 20 | NKX2-4 | 12 | 21324004 | 21326047 | 1.80E-02 |
| 3 | TGM4 | 35 | 44891101 | 44931092 | 1.90E-02 |
| 20 | XRN2 | 30 | 21231941 | 21318463 | 1.90E-02 |
| 13 | FAM123A | 69 | 24640671 | 24643857 | 1.90E-02 |
| 11 | OR52K1 | 57 | 4466706 | 4467651 | 1.90E-02 |
| 10 | C10orf111 | 47 | 15177389 | 15179324 | 1.90E-02 |
| 4 | ELF2 | 25 | 140198320 | 140280056 | 1.90E-02 |
| 11 | CD59 | 66 | 33681131 | 33714601 | 1.90E-02 |
| 2 | TCF7L1 | 149 | 85214244 | 85391016 | 2.00E-02 |
| 6 | PSORS1C1 | 462 | 31190601 | 31215816 | 2.00E-02 |
| 5 | CCDC99 | 30 | 168943215 | 168964359 | 2.00E-02 |
| 1 | TMEM53 | 34 | 44892087 | 44912686 | 2.00E-02 |
| 3 | ATP13A4 | 75 | 194602559 | 194755390 | 2.00E-02 |
| 20 | NKX2-2 | 22 | 21439651 | 21442664 | 2.00E-02 |
| 1 | PCNXL2 | 148 | 231186504 | 231498082 | 2.10E-02 |
| 1 | ACTL8 | 91 | 17954430 | 18026145 | 2.10E-02 |
| 3 | EAF2 | 54 | 123036723 | 123088063 | 2.10E-02 |
| 15 | AGBL1 | 523 | 84486245 | 85373287 | 2.10E-02 |
| 13 | SOHLH2 | 60 | 35640353 | 35686676 | 2.10E-02 |
| 15 | NDNL2 | 38 | 27347649 | 27349309 | 2.20E-02 |
| 6 | ESR1 | 236 | 152053323 | 152466101 | 2.20E-02 |
| 3 | CCK | 52 | 42274321 | 42281399 | 2.20E-02 |
| 7 | HOXA4 | 36 | 27134650 | 27136924 | 2.30E-02 |
| 4 | ING2 | 41 | 184663213 | 184669243 | 2.30E-02 |
| 5 | RPS14 | 26 | 149803984 | 149809512 | 2.30E-02 |
| 1 | CLCA2 | 81 | 86662356 | 86694828 | 2.30E-02 |
| 11 | ANGPTL5 | 36 | 101266614 | 101292463 | 2.30E-02 |
| 11 | EHBP1L1 | 36 | 65100084 | 65116692 | 2.40E-02 |
| 20 | EMILIN3 | 37 | 39422019 | 39428912 | 2.40E-02 |
| 17 | RAI1 | 48 | 17525511 | 17655490 | 2.40E-02 |
| 6 | PPIL1 | 47 | 36930583 | 36950747 | 2.40E-02 |
| 1 | OR2AK2 | 26 | 246195256 | 246196264 | 2.40E-02 |
| 13 | DNAJC3 | 41 | 95127483 | 95241285 | 2.40E-02 |
| 10 | MMP21 | 29 | 127445015 | 127454380 | 2.40E-02 |
| 10 | ADAM12 | 344 | 127693414 | 128067055 | 2.50E-02 |
| 7 | HOXA3 | 36 | 27112333 | 27133164 | 2.50E-02 |
| 10 | ALDH18A1 | 73 | 97355675 | 97406557 | 2.60E-02 |
| 8 | ANGPT2 | 162 | 6344580 | 6408192 | 2.70E-02 |
| 6 | RANBP9 | 51 | 13729708 | 13819775 | 2.70E-02 |
| 4 | BTC | 47 | 75890471 | 75938906 | 2.70E-02 |
| 16 | TMCO7 | 64 | 67435009 | 67676586 | 2.70E-02 |
| 13 | LRCH1 | 135 | 46025303 | 46215731 | 2.70E-02 |
| 19 | CARD8 | 61 | 53403154 | 53444737 | 2.70E-02 |
| 10 | PTER | 96 | 16518972 | 16595742 | 2.70E-02 |
| 6 | NKAIN2 | 466 | 124166767 | 125188485 | 2.90E-02 |
| 10 | RASGEF1A | 42 | 43009989 | 43082373 | 2.90E-02 |
| 10 | RPP38 | 46 | 15179187 | 15186262 | 2.90E-02 |
| 6 | SNAP91 | 59 | 84319331 | 84475495 | 3.00E-02 |
| 11 | KCNK7 | 36 | 65116901 | 65120043 | 3.00E-02 |
| 7 | MKLN1 | 65 | 130663177 | 130831931 | 3.00E-02 |
| 3 | SLC15A2 | 54 | 123095976 | 123143148 | 3.00E-02 |
| 21 | FTCD | 49 | 46380603 | 46399909 | 3.00E-02 |
| 21 | C21orf56 | 44 | 46405497 | 46428729 | 3.00E-02 |
| 1 | SMYD2 | 73 | 212521187 | 212577100 | 3.00E-02 |
| 5 | DIAPH1 | 54 | 140874771 | 140978806 | 3.10E-02 |
| 5 | ZNF608 | 86 | 124000508 | 124108704 | 3.10E-02 |
| 12 | RFX4 | 54 | 105501162 | 105680711 | 3.10E-02 |
| 11 | MMP12 | 46 | 102238673 | 102250922 | 3.10E-02 |
| 20 | RBCK1 | 60 | 336708 | 359610 | 3.10E-02 |
| 19 | EMR3 | 54 | 14591050 | 14646730 | 3.10E-02 |
| 7 | HOXA5 | 41 | 27147520 | 27149812 | 3.20E-02 |
| 19 | TIMM13 | 39 | 2376621 | 2378875 | 3.20E-02 |
| 14 | ACTN1 | 114 | 68410592 | 68515836 | 3.30E-02 |
| 8 | MCPH1 | 286 | 6251528 | 6493434 | 3.30E-02 |
| 7 | HOXA6 | 42 | 27151640 | 27153893 | 3.30E-02 |
| 7 | ZC3HAV1L | 43 | 138360991 | 138371315 | 3.40E-02 |
| 5 | KIF4B | 37 | 154373452 | 154377878 | 3.40E-02 |
| 19 | LILRA2 | 33 | 59777070 | 59790833 | 3.40E-02 |
| 16 | CMIP | 240 | 80036275 | 80302868 | 3.40E-02 |
| 7 | HOXA11 | 42 | 27187300 | 27191360 | 3.50E-02 |
| 5 | MIER3 | 65 | 56251185 | 56283711 | 3.50E-02 |
| 19 | PDE4A | 28 | 10392332 | 10441307 | 3.50E-02 |
| 19 | HAS1 | 64 | 56908176 | 56919033 | 3.50E-02 |
| 17 | MAP2K4 | 47 | 11864859 | 11987776 | 3.50E-02 |
| 7 | HOXA7 | 45 | 27159862 | 27162821 | 3.60E-02 |
| 19 | LAIR2 | 65 | 59705824 | 59713709 | 3.60E-02 |
| 11 | ELF5 | 97 | 34456917 | 34491906 | 3.60E-02 |
| 20 | NRSN2 | 62 | 277892 | 283506 | 3.80E-02 |
| 7 | HOXA9 | 44 | 27168581 | 27171674 | 3.80E-02 |
| 1 | OR2L8 | 33 | 246178782 | 246179721 | 3.80E-02 |
| 10 | UROS | 33 | 127467136 | 127501827 | 3.80E-02 |
| 19 | TYK2 | 38 | 10322203 | 10352248 | 3.90E-02 |
| 17 | NXN | 140 | 649334 | 829760 | 3.90E-02 |
| 22 | TPST2 | 94 | 25251713 | 25316089 | 4.00E-02 |
| 16 | OGFOD1 | 33 | 55042924 | 55068908 | 4.00E-02 |
| 5 | DOCK2 | 302 | 168996870 | 169442959 | 4.00E-02 |
| 17 | RTN4RL1 | 92 | 1784720 | 1874928 | 4.10E-02 |
| 17 | NOS2A | 38 | 23107918 | 23151682 | 4.10E-02 |
| 11 | MAP3K11 | 41 | 65121801 | 65138296 | 4.10E-02 |
| 22 | ZNRF3 | 112 | 27609889 | 27783475 | 4.20E-02 |
| 1 | INADL | 171 | 61980736 | 62402179 | 4.20E-02 |
| 11 | FCHSD2 | 51 | 72225437 | 72530791 | 4.20E-02 |
| 7 | HOXA10 | 47 | 27176734 | 27186401 | 4.30E-02 |
| 5 | GFPT2 | 75 | 179660305 | 179712921 | 4.30E-02 |
| 3 | ARL6IP5 | 40 | 69216779 | 69237929 | 4.30E-02 |
| 20 | TBC1D20 | 47 | 364123 | 391187 | 4.30E-02 |
| 16 | PKD1L2 | 211 | 79691984 | 79811476 | 4.40E-02 |
| 5 | C5orf38 | 63 | 2805261 | 2808511 | 4.40E-02 |
| 3 | RPN1 | 25 | 129821502 | 129852409 | 4.40E-02 |
| 10 | C1QL3 | 60 | 16595747 | 16604010 | 4.40E-02 |
| 5 | CD74 | 34 | 149761392 | 149772525 | 4.50E-02 |
| 2 | OBFC2A | 32 | 192251502 | 192259906 | 4.50E-02 |
| 13 | LHFP | 177 | 38815028 | 39075356 | 4.50E-02 |
| 12 | ATF7IP | 44 | 14409877 | 14542964 | 4.50E-02 |
| 3 | LYZL4 | 58 | 42413578 | 42427069 | 4.70E-02 |
| 9 | TRPM6 | 87 | 76527230 | 76692830 | 4.80E-02 |
| 20 | TRIB3 | 62 | 309307 | 326203 | 4.80E-02 |
| 16 | BBS2 | 65 | 55075798 | 55111696 | 4.80E-02 |
| 5 | HRH2 | 28 | 175042317 | 175044162 | 4.90E-02 |
