# Supplementary material for: Genetics of Obesity Traits: A Bivariate Genome-Wide Association Analysis
Source: Front Genet. 2018 May 16;9:179. doi: 10.3389/fgene.2018.00179 (PMC5964872; doi:10.3389/fgene.2018.00179)
Supplement: Supplementary file 2 [file Table_2.docx]

**Supplementary Table 2**

**Number of significant eQTLs for top 20 genes from VEGAS in the interested single tissue**

| Top 20 gene | Adipose-Subcutaneous (n=385) | Adipose-Visceral (Omentum) (n=313) | Muscle-skeletal (n=491) |
| --- | --- | --- | --- |
| *SLC34A1* | None | None | None |
| *F12* | None | None | 12 |
| *RGS14* | 79 | 13 | 121 |
| *PFN3* | None | None | None |
| *LOC116236* | None | None | None |
| *TP53I13* | 14 | 9 | 15 |
| *MED28* | 496 | 454 | 424 |
| *LAP3* | None | None | None |
| *GNGT2* | None | None | 3 |
| *GIT1* | None | None | 48 |
| *SNX19* | 281 | 307 | 327 |
| *PHOSPHO1* | None | None | 8 |
| *ASPHD2* | 16 | 28 | None |
| *CORO6* | None | 4 | 1 |
| *ANKRD13B* | 511 | 11 | 20 |
| *ABI3* | None | 4 | None |
| *SYT13* | None | None | None |
| *B4GALNT2* | None | None | None |
| *COL16A1* | 7 | None | 2 |
| *HCRTR1* | None | None | None |
